# Usefulness of lung biopsy in critically ill patients undergoing mechanical ventilation

**DOI:** 10.1186/2197-425X-3-S1-A96

**Published:** 2015-10-01

**Authors:** J Marin-Corral, I Oliva, J Leache, L Claverias, V Blazquez, G Moreno, M Magret, M Bodi, C Villavicencio

**Affiliations:** University Joan XXIII Hospital-IISPV-URV, Tarragona, Spain

## Objectives

To evaluate the clinical usefulness of lung biopsy in ventilated patients admitted to an Intensive Care Unit.

## Methods

Retrospective descriptive study that included 16 ventilated patients admitted to the ICU, which underwent a lung biopsy between 2008 and 2013. Demographic data, reason for admission, comorbidities, APACHEII, SOFA, SAPS3, reason for biopsy, clinical data, laboratory results, bronchoalveolar lavage (BAL), radiological pattern and histology, were analyzed. Changes in treatment generated by these results and their impact on prognosis were also analyzed.

## Results

The average age of the population was 60 ( ± 13) years. 68.8% of patients were male. Most patients were admitted for severe CAP (62.5%). The most frequent comorbidities were malignancy and immunosuppression (25%) with APACHE 17 ( ± 5) SAPS3 57 ( ± 14) and SOFA 5 ( ± 2). The main reason for lung biopsy was nonresolving acute respiratory distress syndrome (ARDS, 56.3%). 62.5% of biopsies were opened, 25% were transbronchial and 12.5% were obtained with transthoracic punction. Laboratory results and BAL were inconclusive and the most frequent tomographic pattern was alveolar damage and ground glass (56.2%). The main results of biopsies were idiopathic interstitial disease (56%), tumors (25%) and other processes (19%). In 55.5% of cases the histologic diagnosis led to changes in treatment (start corticosteroids or stop antibiotic). The most common complication related to the procedure was pneumothorax (25%). Intra-ICU mortality was 50% and within patients who died, 75% had neoplastic disease and 25% had interstitial disease.

## Conclusions

Open lung biopsy confirmed the diagnosis in all cases and caused a change in treatment in 50% of them. The most frequent diagnosis was idiopathic interstitial lung disease, which appears to have a better prognosis than neoplasic pathology. In the group of patients with nonresolving ARDS, Lung biopsy could be a useful and safe procedure to guide the treatment and prognosis.

